# The *Arabidopsis thaliana FASCICLIN LIKE ARABINOGALACTAN PROTEIN 4* gene acts synergistically with abscisic acid signalling to control root growth

**DOI:** 10.1093/aob/mcu010

**Published:** 2014-03-05

**Authors:** Georg J. Seifert, Hui Xue, Tuba Acet

**Affiliations:** 1 University of Natural Resources and Life Science, Vienna, Austria; Department of Applied Genetics and Cell Biology, Muthgasse 18, A-1990 Vienna, Austria; 2 Gümüşhane University, School of Health & Nursing, 29100 Gümüşhane, Turkey; 3 Karadeniz Technical University, Science Faculty, Department of Biology, 61080 Trabzon, Turkey

**Keywords:** Fasciclin, arabinogalactan protein, root growth, cell wall, abscisic acid, plant cell wall signalling, *Arabidopsis thaliana*, *At-FLA4*

## Abstract

**Background and Aims:**

The putative *FASCICLIN-LIKE ARABINOGALACTAN PROTEIN 4* (*At-FLA4*) locus of *Arabidopsis thaliana* has previously been shown to be required for the normal growth of wild-type roots in response to moderately elevated salinity. However, the genetic and physiological pathway that connects *At-FLA4* and normal root growth remains to be elucidated.

**Methods:**

The radial swelling phenotype of *At-fla4* was modulated with growth regulators and their inhibitors. The relationship of *At-FLA4* to abscisic acid (ABA) signalling was analysed by probing marker gene expression and the observation of the *At-fla4* phenotype in combination with ABA signalling mutants*.*

**Key Results:**

Application of ABA suppresses the non-redundant role of *At-FLA4* in the salt response. *At-FLA4* positively regulates the response to low ABA concentration in roots and is required for the normal expression of ABA- and abiotic stress-induced genes. The *At-fla4* phenotype is enhanced in the *At-abi4* background, while two genetic suppressors of ABA-induced gene expression are required for salt oversensitivity of *At-fla4*. Salt oversensitivity in *At-fla4* is suppressed by the CYP707A inhibitor abscinazole E2B, and salt oversensitivity in *At-fla4* roots is phenocopied by chemical inhibition of ABA biosynthesis.

**Conclusions:**

The predicted lipid-anchored glycoprotein At-FLA4 positively regulates cell wall biosynthesis and root growth by modulating ABA signalling.

## INTRODUCTION

Among the biopolymers of the plant cell wall, the vast group of hydroxyproline-rich glycoproteins (Showalter *et al.*, 2010) comprises lightly glycosylated proline-rich proteins, moderately glycosylated extensins and highly glycosylated arabinogalactan-proteins (AGPs). The latter, structurally highly diverse family of glycoproteins has been implicated to have a wide range of biological roles; however, there is still an overall lack of understanding of the biophysical and biochemical mode of action of any individual AGP (Seifert and Roberts, 2007; Ellis *et al.*, 2010; Tan *et al.*, 2012).

A sub-group of AGPs, specified by the presence of one or two copies of a fasciclin domain (Fas1), have been termed fasciclin-like AGPs or FLAs (Johnson *et al.*, 2003). The Fas1 domain was named after glycoproteins found in the axonal fascicles of grasshoppers (Bastiani *et al.*, 1987); however, Fas1-containing proteins occur across all phyla (Moody and Williamson, 2013). Fas1 proteins mediate the adhesion between cells and their matrix, e.g. in bacterial biofilms (Moody and Williamson, 2013), or in animal cells, where Fas1 proteins such as periostin and transforming growth factor-β-induced matrix protein (βIG-h3) interact with extracellular matrix receptors of the integrin type (Gillan *et al.*, 2002; Kim *et al.*, 2002). Like many other Fas1-containing proteins, most FLAs localize to the outer leaflet of the plasma membrane via a lipid moiety – specifically a glycosylphosphatidylinositol (GPI) anchor – and associate with putative membrane nanodomains also known as lipid rafts (Borner *et al.*, 2003). The biological roles of FLAs in plant growth and development follow the theme of an involvement in cell wall deposition. The *Arabidopsis thaliana FLA11* and *FLA12* genes are preferentially expressed in secondary cell wall- (SCW) forming cells (Ito *et al.*, 2005; Persson *et al.*, 2005), similar to their orthologues from various other plant species (Dahiya *et al.*, 2006; MacMillan *et al.*, 2010; Liu *et al.*, 2013). Supporting a role in SCWs, the *At-fla11/ At-fla12* double mutant displays a reduction in cellulose content accompanied by reduced tensile strength and tensile modulus of elasticity. This suggests an effect of FLAs both on cellulose deposition and on cell wall matrix integrity (MacMillan *et al.*, 2010). The authors of this paper propose a direct biomechanical role for At-FLA11 and At-FLA12 proteins in the cell wall matrix and also consider a role in signalling that might influence cellulose deposition. Indeed, there is accumulating evidence for FLAs to modulate signalling upstream of cell wall polymer biosynthesis. Overexpression of the cotton orthologue of *At-FLA12,* named *Gh-FLA1* (Liu *et al.*, 2013), in transgenic cotton, results in an increased rate of fibre initiation and elongation and causes an upregulation of a suite of genes related to cell wall biosynthesis including other *FLA* genes, whereas antisense suppression has the opposite effect (Huang *et al.*, 2013). This gain-of-function phenotype shows that FLAs can control the transcriptional programme for cell wall formation, which is best explained by a function in signalling.

The *At-fla4* mutant of *A. thaliana*, also named *salt overly sensitive 5* (*sos5*), highlights a non-redundant role for *At-FLA4* in root growth under salt stress. The root of *At-fla4* shows a drastic reduction of elongation growth combined with radial swelling of the elongation zone. Cell walls appear abnormally thin in *At-fla4*, apparently lacking the middle lamella (Shi *et al.*, 2003). Support for the possibility that *At-FLA4* is involved in a pathway upstream of cell wall deposition comes from the *At-fei1 At-fei2* double mutant, that lacks two leucine-rich repeat receptor-like kinases (LRR-RLKs) resulting in salt oversensitivity just like *At-fla4*, and abnormal cellulose deposition*.* The *At-fei1 At-fei2* double mutant non-additively interacts with *At-fla4*, suggesting that *At-FEI1* and *At-FEI2* act redundantly and *At-FLA4* might act in the same genetic pathway. Moreover, the phenotype of both *At-fei1 At-fei2* and *At-fla4* is suppressed by α-aminoisobutyric acid (AIB), a structural analogue of the ethylene precursor 1-aminocyclopropane-1-carboxylic acid (ACC), and the cytoplasmic domain of FEI2 interacts with several ACC synthase (ACS) proteins, leading to the hypothesis that *At-FLA4* and the *At-FEI* loci might act in a linear genetic pathway that depends on ACC but not on ethylene signalling, upstream of cellulose deposition (Xu *et al.*, 2008).

To better understand the genetic pathway linking *At-FLA4* with salt tolerance and root growth, we used chemical and genetic tools to test the possible involvement of stress-and growth-related signalling pathways. We found that abscisic acid (ABA) suppresses the *At-fla4* mutant phenotype and that ABA signalling is affected by the *At-FLA4* locus. We propose that At-FLA4 might act on ABA signal transduction upstream of cell wall deposition.

## MATERIALS AND METHODS

### Growth conditions and inhibitor treatments

*Arabidopsis thaliana* ecotype Col gl wild type and the *At-fla4* mutant (*sos5-1*) were kindly provided by Jian-Kang Zhu (University of California, Riverside, CA, USA). Mutants *At-cpl1-1*, *At-cpl3-1* and *At-sad1-1* (ecotype C24), *At-abi4-1* and *At-abi5-1* (ecotype Col) were available in our department and, like all mutant combinations, were confirmed by sequencing. Growth conditions were as previously described (Blaukopf *et al.*, 2011). For phenotypic observation, ten seedlings were manually transferred to test media containing standard medium alone or also including the indicated additives, and were examined using a dissecting microscope (Leica EZ4 HD). Unless otherwise stated, figures show representative individuals 48 h after transfer to control or test medium. Abscisic acid, pyrabactin, fluridone [1-methyl-3-phenyl-5-(α,α,α-trifluoro-*m*-tolyl)-4-pyridone] and standard chemicals were obtained from Sigma-Aldrich (Vienna, Austria). Abscinazole E2B was kindly provided by Yasushi Todoroki. Stock solutions were prepared in dimethylsulfoxide (DMSO), except for ABA that was dissolved in 0·01 m NaOH.

### Quantitative real-time PCR (qRT-PCR)

**Fig. 1.**
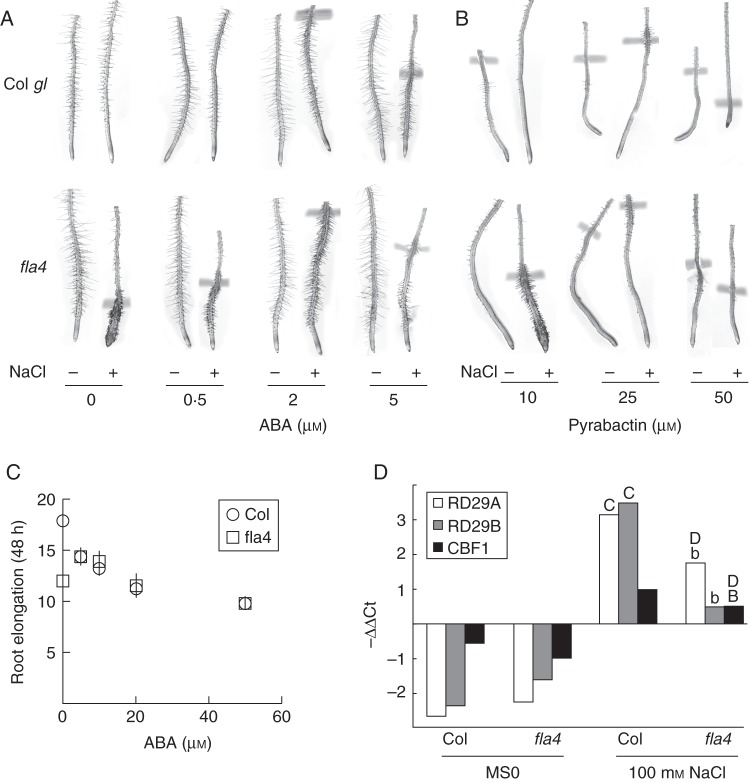
Synergistic effect of *At-FLA4* and ABA signaling. The *At-fla4* root phenotype is suppressed by (A) ABA and (B) pyrabactin. (C) The effect of ABA on root length requires *At-FLA4*. Root length measured after 48 h on media containing different concentrations of ABA (*n* = 20, ±confidence interval, α = 0·01). (D) The effect of salt on the expression of ABA-responsive transcripts in roots depends on *At-FLA4*. The indicated pairs were tested for statistically significant differences, and *t*-test values ≤0·05 and values ≤0·01 are indicated with lower case and uppercase letters, respectively, in the figures. A: Col vs. *At-fla4* on standard medium (MS0), B: Col vs. *At-fla4* on 100 mm NaCl, C: Col MS0 vs. Col NaCl, D: *At-fla4* MS0 vs. *At-fla4* NaCl.

Samples were treated in biological triplicates. For each biological replicate, 150 seedlings were grown on a nylon mesh (20 µm mesh size; Prosep, Belgium) for 5 d and were transferred to standard medium with or without 100 mm NaCl and incubated for 40 min. Roots were removed from the seedlings, frozen in liquid nitrogen, ground in a ball mill (Retsch, Germany) for 2 min and RNA was extracted using peqGOLD Trifast (Peqlab, Germany) according to the manufacturer's instructions. The RNA concentration was measured using a Nano Drop 2000c Spectrophotometer (Thermo Scientific, USA). For each sample, 1 µg of total RNA was reverse-transcribed with oligo(dT) primers using a first-strand cDNA synthesis kit (Thermo Scientific, USA) according to the manufacturer's instructions. Real-time PCR was performed using Solis BioDyne 5 × HOT FIREPol EvaGreen qPCR Mix Plus (no ROX) (Medibena, Austria), and a CFX96^™^ Real-Time PCR Detection System (Bio-Rad, USA) was used for detection. Information on the oligonucleotides used can be found in Supplementary Data Table S1. For real-time PCR, the following program was used: 95·0 °C for 15 min, 40 cycles of 95·0 °C for 10 s, 55·0 °C for 30 s, 72·0 °C for 30 s. Each biological replicate was analysed in technical duplicates. The average technical error was 0·5(±1) Ct values. Technical outliers were identified when the ΔCt between technical replicates was >2·5, and the higher Ct value was removed. The remaining technical replicates were averaged and the ΔCt (Ct test – Ct UBQ5) was calculated. The effect of genotypes and treatments was tested by subjecting the ΔCt values of three biological replicates to a two-sided *t*-test. Test values ≤0·05 and values ≤0·01 are indicated with lower case and uppercase letters, respectively, in the figures as specified in the legend of Fig. 1. To compare the expression levels of different probes graphically, we subtracted the average ΔCt (of three biological replicates) from the series average (average of all samples) to obtain –ΔΔCt. Note that in this way relatively high values indicate relatively high expression levels.

For this study, the relative mRNA levels of *At-FLA4* and six *At-FLA* loci were analysed. The *FLA* loci were selected for their domain structure being similar to that of *At-FLA4* (*At-FLA1*, *At-FLA2*, and *At-FLA8*), for displaying global expression profiles closely correlating with *At-FLA4* (*At-FLA8* and *At-FLA1*; Bjœrn Ost Hansen, unpublished data), for previously showing slight upregulation by ABA [*At-FLA13* and *AtFLA2* (Matsui *et al.*, 2008)] or for generally high expression in roots [*At-FLA2*, *AtFLA7*, *At-FLA8* and *AT-FLA9*; (Johnson *et al.*, 2003)].

## RESULTS

### ABA suppresses the *At-fla4* phenotype and At-FLA4 is required for the ABA-mediated stress response

To define further the physiological process that is controlled by *At-FLA4*, we tested the effect of growth regulators and other bioactive compounds on the *At-fla4* mutant. As previously described (Shi *et al.*, 2003; Xu *et al.*, 2008), *At-fla4* mutants grown in the presence of 100 mm NaCl display a dramatic short root phenotype and radial swelling of the root elongation zone (Fig. 1A). The addition of different growth regulators and compounds affects the *At-fla4* phenotype to varying degrees (data not shown); however, ABA at between 0·5 and 2 µm partially and at 5 µm fully suppresses the NaCl-induced phenotype of *At-fla4* (Fig. 1A). At this concentration, the wild-type and *At-fla4* roots become indistinguishable. Pyrabactin, a synthetic inhibitor of seed germination (Zhao *et al.*, 2007) acting as a selective agonist of ABA perception (Park *et al.*, 2009), suppresses the *At-fla4* root phenotype at a concentration of 25 µm (Fig. 1B). Moreover, pyrabactin inhibits root elongation and root hair growth in the presence and absence of NaCl; however, *At-fla4* appears less sensitive to this inhibition than the wild type. *At-FLA4* is not only necessary for normal root growth on 100 mm NaCl- or 4 % sucrose-containing medium (Xu *et al.*, 2008). Also on NaCl-free medium containing 1 % sucrose, *At-fla4* roots are significantly (*P* < 0·001) shorter than those of the wild type (Fig. 1C) and more radially expanded compared with the wild type, giving the appearance of relatively dense root hairs initiating closer to the root tip (Fig. 1A). Application of ABA causes a dose-dependent decrease of root elongation in the wild type. By contrast, the elongation of *At-fla4* roots is not negatively affected by up to 10 µm ABA; in fact, between zero and 5 µm ABA, there is a significant (*P* < 0·001) increase in *At-fla4* root length. On the one hand this means that the suppression of the *At-fla4* phenotype by ABA is not restricted to high salt treatment. On the other hand, this observation also suggests that *At-FLA4* acts in the inhibition of root elongation by relatively low doses of ABA.

The mRNA levels of the abiotic stress-induced, ABA-regulated genes *At-RD29A*, *At-RD29B* and *At-CBF1* in *At-fla4* are not significantly different from those of the wild type 40 min after transfer to NaCl-free control medium. After 40 min treatment with 100 mm NaCl, the three marker genes are upregulated in both genotypes. However, the transcript levels of all three genes are significantly lower in *At-fla4* compared with the wild type (Fig. 1C). In summary, ABA suppresses the role of *At-FLA4* for root growth and salt tolerance and *At-FLA4* positively regulates responses to ABA and salt in roots.

### The *At-fla4* phenotype is modulated by ABA response mutants

**Fig. 2.**
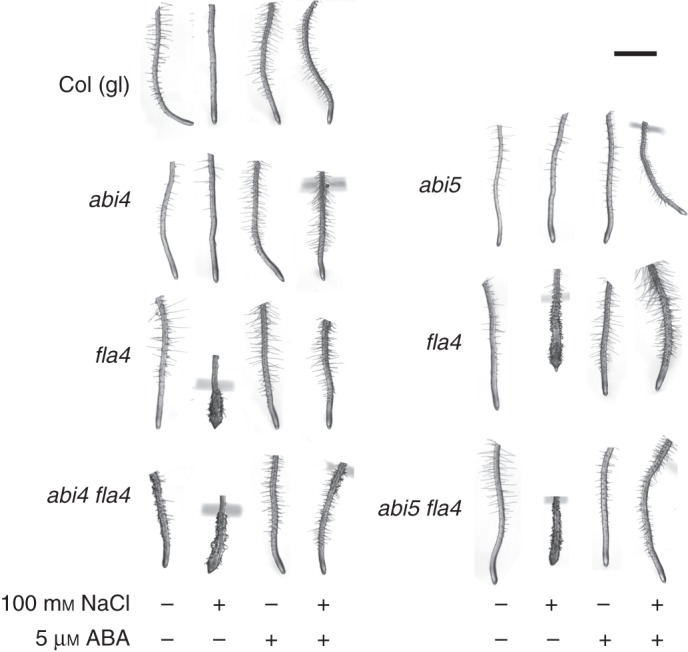
Genetic interaction between *At-FLA4* and positive ABA response regulators *At-ABI4* and *At-ABI5*. Note that the *abi4 fla4* double mutant shows abnormal root growth on NaCl-free medium. Scale bar = 1 mm.

To demonstrate that the role of *At-FLA4* in salt tolerance and root growth is mediated by genetic regulators of the ABA response, we isolated double mutants between *At-fla4* and ABA-insensitive and ABA-oversensitive mutants. The *At-ABI4* and *At-ABI5* loci encode positive transcriptional regulators of the ABA response acting during seed germination and other ABA-dependent processes (Finkelstein, 1994; Dai *et al.*, 2013; Wind *et al.*, 2013). However, *At-abi4 At-fla4* and *At-abi5 At-fla4* roots are fully responsive to 5 µM ABA with respect to the suppression of the *At-fla4* phenotype (Fig. 2), which means that this ABA effect does not depend on the function of *At-ABI4* or *At-ABI5*. Neither *At-abi4* nor *At-abi5* displays obvious root growth phenotypes on NaCl-containing or control medium. By contrast, the *At-abi4 At-fla4* double mutant appears phenotypically abnormal on NaCl-free medium, displaying a relatively short root and epidermal bulging (Fig. 2). This suggests a genetic synergy of *At-FLA4* and *At-ABI4* in root growth.

**Fig. 3.**
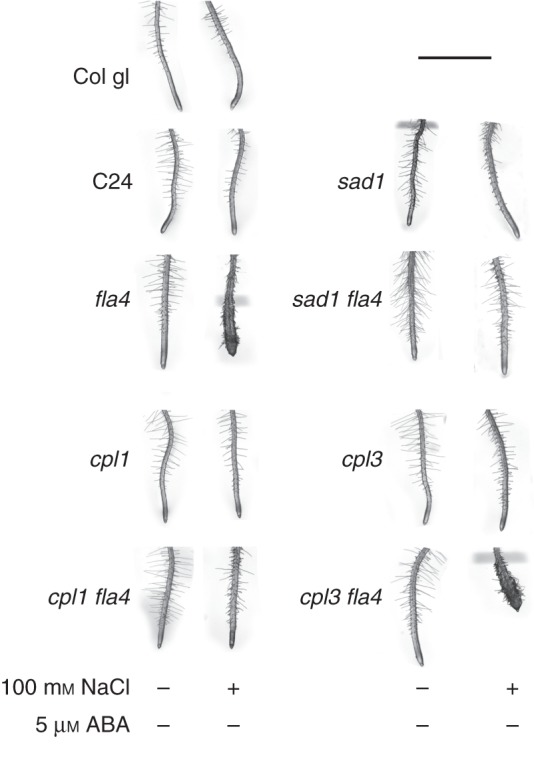
Genetic interaction between *At-FLA4* and ABA repressors *At-CPL1*, *At-CPL3* and *At-SAD1*. Conditions are the same as for Fig. 2. Note that on 100 mm NaCl-containing medium, the *At-cpl1 At-fla4* and the *At-sad1 At-fla4* double mutants are comparable with the wild type, while the *At-cpl3 At-fla4* double mutant is comparable with the *At-fla4* single mutant. Scale bar = 2 mm.

The *C-TERMINAL DOMAIN PHOSPHATASE LOCI 1* and *-3* [*At-CPL1* (Koiwa *et al.*, 2002; Xiong *et al.*, 2002*a*) and *At-CPL3* (Koiwa *et al.*, 2002)] and *SUPERSENSITIVE TO ABA AND DROUGHT* [*At-SAD1* (Xiong *et al.*, 2001)] encode negative regulators of ABA-induced mRNA species. In the presence and absence of 100 mm NaCl, root morphology in double mutants *At-cpl1 At-fla4* and *At-sad1 At-fla4* is comparable with that of the wild type (Fig. 3). By contrast, the *At-cpl3 At-fla4* double mutant displays salt oversensitivity comparable with that of the *At-fla4* single mutant. This indicates that *At-CPL1* and *At-SAD1* are required for the genetic regulation of salt sensitivity by *At-FLA4*. Taken together, the non-redundant role of *At-FLA4* in root growth is enhanced by the positive ABA regulator *At-ABI4* and suppressed by the negative ABA regulators *At-CPL1* and *At-SAD1*.

### Involvement of ABA metabolism in the role of *At-FLA4* in root growth

**Fig. 4.**
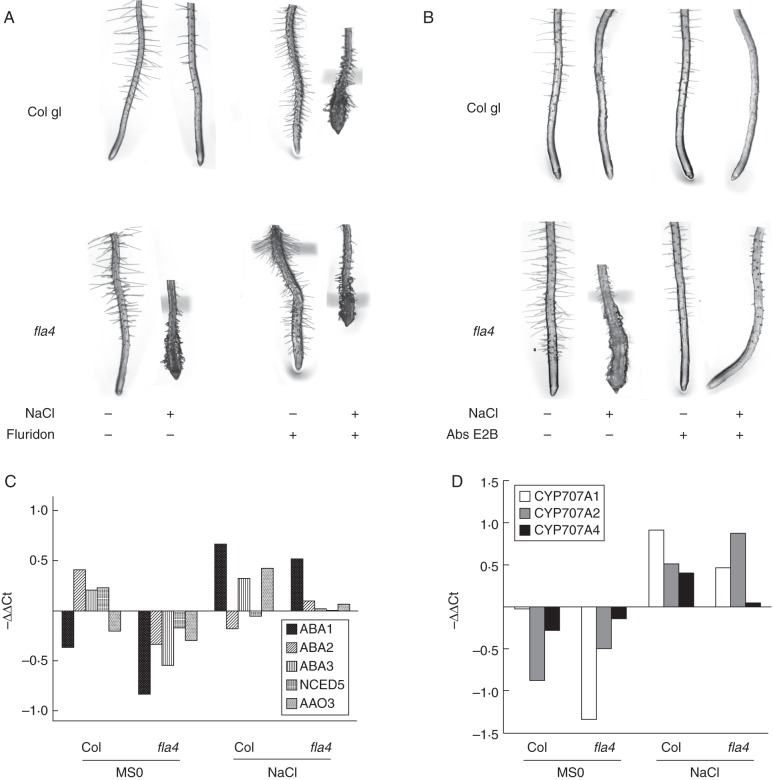
Interaction of *At-FLA4* with ABA metabolism. (A) Inhibition of ABA biosynthesis with fluridone phenocopies the *At-fla4* phenotype. (B) Inhibition of ABA catabolism with abscinazole E2B suppresses the *At-fla4* phenotype. Expression of genes involved in ABA (C) biosynthesis and (D) catabolism is not significantly different between Col and *At-fla4*.

For the investigation of the potential interaction between *At-FLA4* and ABA metabolism, we used fluridone to inhibit carotenoid biosynthesis, an early step in ABA biosynthesis. Fluridone in the absence of NaCl reduces root elongation and enhances radial expansion in the wild type and to a visibly higher degree in *At-fla4* (Fig. 4A). In combination with NaCl, fluridone induces dramatic root swelling in the wild type that strikingly resembles the *At-fla4* phenotype. However, under the same conditions, *At-fla4* mutants show a more severe phenotype compared with the wild type, suggesting that *At-FLA4* action might be synergistic with but independent of ABA biosynthesis (Fig. 4A). The specific CYP707A inhibitor abscinazole E2B that interferes with ABA 8'-hydroxylase (Okazaki *et al.*, 2012) suppresses salt oversensitivity in *At-fla4.* The subtle *At-fla4* root phenotype on NaCl-free medium is also suppressed by this compound (Fig. 4B). Hence with respect to *At-FLA4* function in roots, ABA biosynthesis has a synergistic effect while ABA catabolism has an antagonistic effect.

To investigate the possible involvement of *At-FLA4* as a regulator of ABA metabolism, we measured the relative transcript level of genes involved in this process (Nambara and Marion-Poll, 2005) in *At-fla4* mutants compared with wild-type roots before and after 40 min exposure to 100 mm NaCl. None of five ABA biosynthetic loci (*At-ABA1*, *At-ABA2*, *At-ABA3*, *At-NCED5* and *At-AAO3*) or three ABA catabolic loci (*At-CYP707A1*, *At-CYP707A2* and *At-CYP707A4*) displays a significant difference between *At-fla4* mutant and wild-type roots. However, the transcript level of all five tested biosynthetic loci is reduced in the mutant compared with the wild type under salt-free conditions, and after salt treatment *At-ABA1*, *At-ABA3* and *At-AAO3* are reduced in *At-fla4* compared with the wild type (Fig. 4C). By contrast, two of the three tested ABA catabolic loci are insignificantly upregulated in *At-fla4* compared with the wild type under salt-free conditions. After salt exposure, only *At-CYP707A2* is upregulated, while *At-CYP707A1* and *-4* are downregulated in *At-fla4* compared with the wild type (Fig. 4D). Taken together, we do not observe a clear effect of *At-FLA4* on ABA metabolism at the individual transcript level. Nevertheless, the locus might contribute to achieving the appropriate balance between ABA catabolic and biosynthetic gene expression. Consistent with the possibility that *At-FLA4* stimulates ABA biosynthesis and downregulates ABA catabolism, we observe that *At-fla4* loss of function and ABA biosynthesis inhibition are synergistic, and chemical inhibition of ABA catabolism phenotypically suppresses *At-fla4*.

### ABA does not affect *FLA* transcript levels in roots

**Fig. 5.**
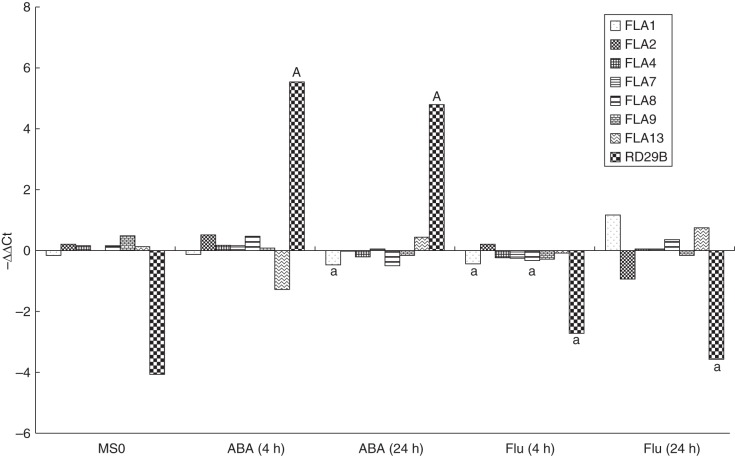
Effect of ABA (5 µM) and fluridone (5 µm) on the transcript level of selected *At-FLA* genes and *At-RD29B*. To mark significant differences between standard medium (MS0) and media containing ABA or fluridone, *t*-test values ≤ 0·05 and ≤0·01 are indicated with lower case a and uppercase A, respectively.

To test the effect of ABA on *FLA4* expression, we compared the level of *At-FLA4* transcript in roots treated with 5 µm ABA or 5 µm fluridone for 4 and 24 h with untreated controls. While *At-RD29B* mRNA is significantly (*P* < 0·01) upregulated by ABA treatment, the level of *At-FLA4* transcript barely varies between the treatments. To test whether other *FLA* loci might be induced by ABA, to compensate for the loss of *At-FLA4*, we selected *At-FLA1*, *At-FLA2*, *At-FLA7*, *At-FLA8*, *At-FLA9* and *At-FLA13* genes based on considerations outlined in the Materials and Methods. Compared with *At-RD29B* that displays a difference of approx. 10 Ct units between untreated and ABA-treated roots, the fluctuations of the selected *FLA* genes are very moderate, the largest albeit statistically insignificant difference of 1·29 Ct units seen after 4 h ABA treatment being shown by *At-FLA13* (Fig. 5). Of the seven tested *FLA* genes, *At-FLA1* shows the only statistically significant (*P* < 0·05) difference between ABA-treated and non-treated roots. With a value of 0·29 Ct units, this corresponds to a very subtle, probably irrelevant decrease. Likewise, fluridone treatment causes only weak alterations in the expression level in any of the tested *FLA* genes, with *At-FLA1* and *At-FLA8* being expressed at a slightly lower level after 4 h of treatment. Hence under our test conditions, ABA does not influence the RNA level of *At-FLA1*, *At-FLA2*, *AtFLA4*, *At-FLA7*, *At-FLA8*, *At-FLA9* and *At-FLA13*.

## DISCUSSION

### The actions of *At-FLA4* and ABA are synergistic

**Fig. 6.**
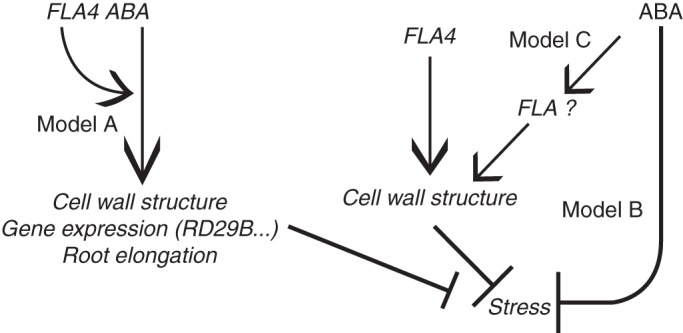
Genetic model for the interaction of *At-FLA4* with ABA. For an explanation, see the Discussion.

In this study, multiple lines of evidence support the view that *At-FLA4* acts in synergy with ABA signalling (Fig. 6). (1) Externally applied ABA suppresses *At-fla4*, both its salt-oversensitive phenotype and its root elongation phenotype, under salt-free conditions. (2) Root elongation in the *At-fla4* mutant shows reduced sensitivity towards inhibition by ABA, and ABA-regulated transcripts are downregulated in salt-treated *At-fla4* compared with the wild type. (3) The ABA-insensitive *At-abi4-1* mutation enhances *At-fla4* under NaCl-free conditions and the ABA-oversensitive *At-cpl1-1* and *At-sad1-1* alleles suppress *At-fla4*. (4) The *At-fla4* phenotype is phenocopied by inhibition of ABA biosynthesis and suppressed by the inhibition of ABA degradation.

The central observation presented in this study is the phenotypic suppression of *At-fla4* by ABA and pyrabactin. The latter is a selective agonist of the PYR1/PYL-type ABA receptor and does not interact with other potential ABA receptor types (Park *et al.*, 2009; Klingler *et al.*, 2010). The ‘core signalling pathway’ consists of PYRABACTIN RESISTANT 1/PYR1 LIKE (*At-*PYR1/PYL) proteins that are induced by ABA to bind to type 2C protein phosphatases (PP2Cs) such as *At-*ABI1, thereby releasing them from inhibiting SNF1-related protein kinases (SnRK2s). The de-repressed SnRK2s in turn phosphorylate and activate a number of substrates including membrane channels and transcription factors such as *At-*ABI4 and *At-*ABI5 (Hubbard *et al.*, 2010). Consequently, activation of the ABA ‘core signalling pathway’ is sufficient to suppress the *At-fla4* salt-oversensitive phenotype. Although the effect of *At-ABI4* on ABA sensitivity of the root is only partial (data not shown), the enhancement of the *At-fla4* phenotype on NaCl-free medium suggests that *At-FLA4* and *At-ABI4* are synergistic. Consistently, two negative regulators of ABA-induced gene expression, *At-CPL1* and *At-SAD1*, are antagonistic with respect to *At-FLA4*. The *At-CPL1*, *At-CPL3* and *At-SAD1* loci were identified as negative regulators of the stress response and ABA signalling, and their loss-of-function mutant alleles display overaccumulation of *At-RD29A* transcript (Xiong *et al.*, 2001, 2002*a*; Koiwa *et al.*, 2002). While various CPLs might negatively regulate RNA polymerase II by dephosphorylating its C-terminus, *At-CPL1* is essential for normal micro RNA biogenesis, and genetic defects in this process lead to ABA hypersensitivity (Manavella *et al.*, 2012). Another ABA-hypersensitive locus that antagonizes *At-FLA4* is *At-SAD1*, encoding the SM-like 5 protein [LSM5 (Xiong *et al.*, 2001)]. The *At-*SAD1/LSM5 protein is a component of both the LSM1–LSM7 and the LSM2–LSM8 complex that act in mRNA degradation and pre-mRNA splicing, respectively (Perea-Resa *et al.*, 2012). The suppression of *At-fla4* by *At-cpl1* and *At-sad1* suggests that sufficient accumulation of ABA-induced mRNA species is required for *At-FLA4* to fulfil its role. The lack of suppression of *At-fla4* by *At-cpl3* might be explained by genetic redundancy of *At-CPL3* in the root. It might also indicate that the set of mRNAs repressed by *At-CPL3* is not identical to the set of *At-CPL1*- and *At-SAD1*-regulated mRNAs that are downregulated in salt-treated *At-fla4* and which might be involved in the *At-fla4* phenotype.

The reduced level of *At-RD29A*, *At-RD29B* and *At-CBF1* in the *At-fla4* background upon salt treatment is consistent with a positive role for *At-FLA4* in ABA signalling because normal induction of these transcripts depends on ABA signalling (Xiong *et al.*, 2002*b*; Knight *et al.*, 2004). Initially, we expected an increased level of abiotic stress response in the *At-fla4* mutant compared with the wild type. After genetic and chemical inhibition of cellulose biosynthesis, transcriptional signatures indicative of jasmonic acid- and ethylene-regulated biotic and wounding stress can be observed (Ellis and Turner, 2001; Guan and Nothnagel, 2004; Hamann *et al.*, 2009). Such responses might, however, result from lesions in a constitutive mutant or be caused by long-term inhibitor treatment. By analysing roots exposed to NaCl for 40 min, we tested a time point when root morphology was not visibly damaged but when the transcriptional salt stress response was already activated, as indicated by induction of *At-RD29A*, *At-RD29B* and *At-CBF1*. We also analysed several other genes that respond to cell wall inhibitors such as isoxaben (Hamann *et al.*, 2009) and β-glucosyl Yariv reagent (Guan and Nothnagel, 2004). *OXIDATIVE SIGNAL INDUCIBLE 1* (Rentel *et al.*, 2004) and *RESPIRATORY BURST OXYDASE HOMOLOG D* (Ma *et al.*, 2012) and several other genes were not significantly changed by 40 min NaCl treatment or between the mutant and wild type. *At-ACS6*, *At-MYB15*, *At-MUR4* and *At3g11280* were induced by NaCl; however, none of these loci showed a significant effect of *At-fla4* (data not shown). By contrast, all tested ABA-dependent genes displayed a significant repression in *At-fla4,* suggesting that the effect of *At-FLA4* on stress signalling might be specific for ABA.

Fluridone inhibits the biosynthesis of carotenoid precursors for ABA (Nambara and Marion-Poll, 2005) and it causes salt oversensitivity closely resembling that of *At-fla4*. Interestingly, root elongation defects were previously observed in *At-aba1* and *At-aba2* mutants (Barrero *et al.*, 2005; Lin *et al.*, 2007); however, residual ABA that is detected in *aba* mutants suggests the existence of an alternative ABA biosynthesis pathway supporting essential growth processes (Barrero *et al.*, 2005). Fluridone might more fully deplete the endogenous ABA, leading to the observed growth defects. The apparent additive effect of *At-fla4* and fluridone, however, indicates that although *At-FLA4* and ABA biosynthesis are synergistic with respect to root growth and salt sensitivity, they act in separate pathways. Accordingly, *At-FLA4* does not act by affecting ABA biosynthesis. However, this hypothesis requires genetic confirmation to exclude side effects of fluridone.

The suppression of *At-fla4* by the CYP707A-specific inhibitor abscinazole E2B (Okazaki *et al.*, 2012) is consistent with our view that ABA and *At-FLA4* are synergistic, because inhibition of CYP707A that functions as an ABA 8'-hydroxylase *in planta* is expected to block the main catabolic pathway for ABA (Nambara and Marion-Poll, 2005). Our observation indicates that in *At-fla4*, the intracellular ABA level or sensitivity are below a threshold level to counterbalance salt stress sufficiently and that the ABA level is raised by blocking ABA degradation. However, the expression level of seven ABA biosynthetic genes and three catabolic genes is not significantly different between *At-fla4* and the wild type, which argues against the alteration of the endogenous ABA level dependent on *At-FLA4*. Nevertheless, we cannot exclude cell type-specific alterations of ABA metabolism in *At-fla4*.

### How does ABA suppress *At-fla4*?

Taken together, we suggest that *At-FLA4* and ABA signalling act in genetic synergy, leading to suppression of *At-fla4* by ABA and to a reduced ABA response in *At-fla4*. We envisage three alternative models for this effect. First, *At-FLA4* might positively modulate an ABA-dependent effect on root elongation, gene expression and cell wall structure (Fig. 6, Model A). According to this view, the loss of *At-FLA4* function is expected to compromise the final output of ABA signalling. External ABA supply corrects this defect by exceeding a threshold required for normal cell growth under salt-free conditions and for tolerance of salt. This hypothesis also predicts that besides known ABA responses such as *At-RD29A* expression and inhibition of root elongation, processes required for cell wall structural integrity exist downstream of *At-FLA4*-modulated ABA signalling. This prediction is consistent with our observation that depletion of ABA by fluridone phenocopies *At-fla4* under salt stress.

The two alternative hypotheses suggest that *At-FLA4* acts primarily in an ABA-independent pathway upstream of cell wall structure (Fig. 6, Models B and C). Consequently, the primary effect of the loss of *At-FLA4* function is a damaged cell wall subsequently leading to cellular stress responsible for the apparent phenotypic changes. In one variant of the model, ABA counteracts the indirect consequences of cell wall damage initiated by the *At-fla4* mutation (Fig. 6, Model B), possibly by the induction of salt export (Shi and Zhu, 2002) or antioxidants (Ding *et al.*, 2013). In a second variant, ABA activates genes that make *At-FLA4* redundant. The induction of alternative *FLA* genes by ABA might provide a simple mechanism for this scenario (Fig. 6, Model C). However, although many *FLA* loci are differentially regulated by ABA, in most cases a repression of FLA mRNA by ABA is observed. In one study, *At-FLA1*, *At-FLA2* and *At-FLA8* mRNAs are downregulated by ABA (Johnson *et al.*, 2003). Likewise, the genetic manipulation of ABA levels affects the transcript levels of 11 of the 21 *FLA* genes in the *A. thaliana* genome (Okamoto *et al.*, 2010) and also under these conditions *FLA* genes are exclusively downregulated by ABA. Finally, in our study that focuses on roots, there is no apparent response of a set of seven *At-FLA* loci to ABA application and depletion. Besides the possibility of post-transcriptional regulation that we have not tested, these observations make an ABA-induced suppression of *At-fla4* by alternative FLAs unlikely.

At present we cannot exclude any of the proposed scenarios of how *At-fla4* and ABA might interact; however, neither Model C nor Model B (Fig. 6) accounts for the reduced level of ABA-regulated transcripts in *At-fla4* or for its reduced response to low concentrations of ABA, whereas Model A explains both the suppression of the *At-fla4* phenotype by ABA and the suppression of ABA responses in *At-fla4*. To support Model A further, we envisage a novel mechanism of how FLA proteins might influence ABA signalling.

### How FLAs might modulate ABA signalling

To explain our observations, we favour a model in which *At-FLA4* positively modulates ABA signalling (Fig. 6, Model A) and hypothesize how the At-FLA4 protein might be mechanistically involved in this process. In one plausible scenario, *At-FLA4* might regulate the early events in the ‘core signalling pathway’ of ABA signal transduction. The crucial step in ABA perception requires the suppressive effect of PP2Cs on SnRK2s to be released by the ABA-bound form of PYR1/PYL proteins (Hubbard *et al.*, 2010). We speculate that the physical interaction between PP2Cs and one or more of their partners in this complex might be modulated by factors in addition to ABA. Intriguingly, the PP2C At-ABI1 and another PP2C protein have recently been identified in detergent-resistant lipid rafts (Demir *et al.*, 2013). FLA proteins are typically associated with the outer face of lipid rafts in the plasma membrane via their GPI anchor (Borner *et al.*, 2003; Kierszniowska *et al.*, 2009; Demir *et al.*, 2013). Hence FLAs and ABI1 might assemble in the same plasma membrane sub-domain. However, if *At-FLA4* indeed physically interacts with the cytosolic At-ABI1, such an interaction can only be indirect. A protein group that is in a position to connect At-FLA4 and At-ABI1 physically are receptor-like kinases, many of which have recently been detected in lipid rafts (Shahollari *et al.*, 2004; Demir *et al.*, 2013). Furthermore, the At-FEI1 and At-FEI2 LRR-RLKs have been speculated to bind to At-FLA4 and mediate its action (Xu *et al.*, 2008). Interestingly, both At-FEI2 and At-ABI1 physically interact with different forms of ACS. At-ABI1 interacts with At-ACS2 and At-ACS6 (Ludwikow *et al.*, 2009), while At-FEI2 interacts with At-ACS5 and At-ACS9 but not with At-ACS2 in yeast two-hybrid assays (Xu *et al.*, 2008). Although such assays do not necessarily reflect *in vivo* binding, ACS isoforms forming a complex with At-ABI1 and At-FEI2 in principle might physically link At-FLA4 and ABA signalling in this speculative scenario. Thereby, this putative complex might connect ABA signalling with the previously postulated ethylene-independent action of ACC on elongation growth (Xu *et al.*, 2008; Tsang *et al.*, 2011).

At present, direct evidence for a physical interaction between FLAs and receptor-like proteins on the one hand and between ABI1 and membrane proteins on the other hand is lacking. The physical interaction between Fas1 domain-containing mammalian glycoproteins βIG-h3 and periostin with integrin-type cell surface receptors is revealed in adhesion assays using intact cells spreading on coated glass cover slips (Kim *et al.*, 2000, 2002; Gillan *et al.*, 2002). Such cell adhesion assays are not available for plant cells, but other approaches such as co-immunoprecipitation and Förster resonance energy transfer (FRET) might be more promising to identify physical interactors of FLA proteins.

Overall, we conclude that *At-FLA4* regulates normal root growth via an ABA-depedent signalling pathway that might be upstream of cell wall biosynthesis.

## SUPPLEMENTARY DATA

Supplementary data are available online at www.aob.oxfordjournals.org and consist of Table S1: list of oligonucleotides used for qRT-PCR.

Supplementary Data
