# Supplementary material for: The Arabidopsis thaliana FASCICLIN LIKE ARABINOGALACTAN PROTEIN 4 gene acts synergistically with abscisic acid signalling to control root growth
Source: Ann Bot. 2014 Mar 5;114(6):1125–33. doi: 10.1093/aob/mcu010 (PMC4195540; doi:10.1093/aob/mcu010)
Supplement: Supplementary Data [file supp_114_6_1125__index.html]

The Arabidopsis thaliana FASCICLIN LIKE ARABINOGALACTAN PROTEIN 4 gene acts synergistically with abscisic acid signalling to control root growth — Supplementary Data 

# The *Arabidopsis thaliana FASCICLIN LIKE ARABINOGALACTAN PROTEIN 4* gene acts synergistically with abscisic acid signalling to control root growth

## Supplementary Data

Supplementary Data

**Files in this Data Supplement:**

- Supplementary Data - Pdf file
