# Supplementary material for: The Arabidopsis thaliana FASCICLIN LIKE ARABINOGALACTAN PROTEIN 4 gene acts synergistically with abscisic acid signalling to control root growth
Source: Ann Bot. 2014 Mar 5;114(6):1125–33. doi: 10.1093/aob/mcu010 (PMC4195540; doi:10.1093/aob/mcu010)
Supplement: Supplementary Data [file supp_mcu010_mcu010supp.pdf]

SUPPLEMENTARY DATA

Table S1. Oligonucleotides used for QRT-PCR.

Gene name	Locus name	f-oligo	r-oligo
AtRD29A	*At5g52310*	aagttactgatcccaccaaagaagaaac	tttcctcccaacggagctcctaaac
AtRD29B	*At5g52300*	tccggtttacgaaaaagtcaaagaaac	aatccgaaaaccccatagtcccaac
AtCBF1	*At4g25490*	atgcgccaaggatatccaaaaagc	ccaaagcgacacgtcaccatctc
AtABA1	*At5g67030*	gtggtttgaagatgacgatgc	gattactttcaccctaaacgcc
AtABA2	*At1g52340*	gcattgttcgtctgttccac	atcttccactctaacatcgcc
AtABA3	*At1g16540*	agagtcctggtttgtgcag	tctcgtatctcaaaagcgttcc
AtNCED5	*At1g30100*	tccaaaccccataattcccg	cataccgtcaccgtcgaatag
AtAAO3	*At2g27150*	ctatggagttggagtcagcg	tttccgagcttccctaatgg
AtCYP707A1	*At4g19230*	tcacattcagagaagctgtcg	gtcattctacacttcgatctccg
AtCYP707A2	*At2g29090*	aaatgccactgaccactagg	atctccaatcacttcccatctg
AtCYP707A4	*At3g19270*	tcagagaagcagtggttgatg	acgaaatgtagcgggaagac
AtFLA1	*At5g55730*	acaaacacacctcgccgacgaaatc	agatgttatacggaacttcttcaatag
AtFLA2	*At4g12730*	aaaaggcggtaaagtcgcttttggtg	ttgttcgccggagaaagggatttg
AtFLA4	*At3g46550*	taaaaataaataaagccctaatcgaagg	atcacagtcttcgtccccaccgactc
AtFLA7	*At2g04780*	tccagtgctgccaccgacac	agtattgaccaccagcaaaggtg
AtFLA8	*At2g45470*	aaacatcaccggattactcgaaaaag	agcaacgccggtgtggagaataac
AtFLA9	*At1g03870*	tcaagcttctggccgagacaac	agaaatttcaaacataagacaataag
AtFLA13	*At5g44130*	acatcacggcaatcctcgaaaaag	acgacaccagtagatacattgactt
